# Study on the Thermal Properties and Reliability of Liquid Metal/Copper-Coated Diamond Composite Thermal Interface Materials

**DOI:** 10.3390/nano16211340

**Published:** 2026-10-22

**Authors:** Zimeng Chen, Bo Wang, Zhanglong Ke, Wei Huang, Yubing Gong, Siliang He, Kailin Pan

**Affiliations:** 1 Guangxi Key Laboratory of Advanced Packaging and System Integration, School of Mechanical and Electrical Engineering, Guilin University of Electronic Technology, Guilin 541004, China; zimengc@163.com (Z.C.); 17827775516@163.com (Z.K.);; 2 Research Institute of Sun Yat-sen University in Huizhou, Huizhou 516081, China

**Keywords:** liquid metal, thermal interface materials, copper-coated diamond, reliability, finite element simulation

## Abstract

As the integration and power density of high-power electronic devices continue to rise, efficient thermal management has become a key challenge in ensuring their performance and reliability. In this study, liquid metal gallium was used as the matrix and copper-coated diamond (CCD) as the reinforcing phase. Using a mechanical mixing method, liquid metal/copper-coated diamond (LM-CCD) composite thermal interface materials with different filler contents (10–60 vol%) were prepared, and their microstructure, thermal conductivity, and high-temperature operational reliability were systematically investigated. The results showed that when the CCD content is 50 vol%, a complete, interconnecting thermal conduction network forms within the composite, with thermal conductivity peaking at 86.9 W/(m·K)—approximately three times that of pure gallium (~29 W/(m·K)). COMSOL Multiphysics 6.2 finite element simulations confirmed that the thermal conduction framework formed by high CCD content effectively reduces heat accumulation. However, high-temperature aging tests at 125 °C revealed a synergistic effect between the formation of the CuGa_2_ intermetallic compound (IMC) via interfacial reactions and oxidation reactions, resulting in microstructural degradation and a drop in thermal conductivity to approximately 49.3% of its initial value after 240 h. After 336 h of aging, the thermal conductivity decreased further to approximately 27 W/(m·K), indicating that the thermal conductivity enhancement provided by CCD had been lost under these accelerated aging conditions. In addition, actual CPU thermal testing has shown that, compared to commercial thermal paste (~5 W/(m·K)), the LM-CCD composite material can reduce the chip’s steady-state temperature by approximately 14 °C. This represents a 4 °C reduction compared to pure gallium. Through combined experimental and theoretical analyses, this study provides a basis for understanding the degradation mechanisms of liquid-metal thermal interface materials and for designing more reliable TIMs through interface modification or oxidation-resistant encapsulation.

## 1. Introduction

The continuous push for higher integration and miniaturization in high-power electronics has led to escalating chip power densities, thereby imposing increasingly stringent requirements on thermal management [1]. Thermal interface materials serve as critical fillers for microscopic gaps between chips and heat sinks. Their thermal conductivity directly governs the thermal efficiency of the packaging system and the operating temperature of the device [2]. Traditional polymer-based thermal interface materials (TIMs), including thermal grease, thermal pads, and phase-change materials, typically exhibit thermal conductivities ranging from 0.4 to 5 W/(m·K) [3,4], which are no longer sufficient to meet increasingly stringent thermal management requirements [5,6]. Although metal-based TIMs (such as indium and tin) have high thermal conductivity [7], they generally suffer from issues such as high hardness, poor flexibility, and high interfacial thermal resistance [2,7]. Although carbon-based TIMs (such as graphene, carbon nanotubes, and their composites) exhibit excellent intrinsic thermal conductivity (the in-plane thermal conductivity of monolayer graphene can reach 5300 W/(m·K)) [5], their out-of-plane thermal conductivity is generally low, and their fabrication processes are complex and costly [8,9]. Against this backdrop, gallium-based liquid metals (LMs) have attracted significant attention due to their unique physicochemical properties [10,11]. The intrinsic thermal conductivity of pure gallium is ~29 W/(m·K), while its melting point is around 29.76 °C [12]; it combines good fluidity with deformability, enabling it to effectively fill microscopic irregularities at interfaces and achieve extremely low interfacial contact thermal resistance [13,14]. Furthermore, liquid metal possesses a high boiling point and good thermal stability [11,15]. However, its reliability in actual operating environments is significantly affected by interfacial reactions and oxidation behavior. First, it has high surface tension (approximately 0.708 N/m) and poor wettability on most solid surfaces; when exposed to air, a dense Ga_2_O_3_ oxide film forms on the surface of gallium droplets, making it difficult to spread evenly [16]. Second, liquid metals exhibit excellent fluidity, making them prone to “pumping out” and “leaking” during operation, which may pose a risk of circuit short-circuits [17,18]. Additionally, gallium-based liquid metals are corrosive to common heat-sink metals, e.g., copper and aluminum; prolonged contact with aluminum results in embrittlement of the liquid metal, which seriously undermines the long-term reliability of the devices [15,19].

To overcome the aforementioned limitations, researchers have developed performance optimization strategies focusing on filler reinforcement and interfacial modification. In terms of filler reinforcement, introducing high-thermal-conductivity particles into the liquid metal matrix can significantly enhance the thermal conductivity of the composite material [20,21]. Diamond is an extremely hard material, widely recognised for its abrasive properties. However, diamond also possesses the highest intrinsic thermal conductivity of any bulk material (approximately 2000–2200 W/(m·K)), making it a highly attractive reinforcing filler for thermal management composites. A great deal of research has focused on incorporating diamond particles into polymer or metal matrices to enhance thermal conductivity. When used in thermal interface materials, the sharp edges of diamond particles may cause mechanical wear or scratching to mating surfaces under clamping pressure, thereby increasing surface roughness and interfacial thermal resistance. It is therefore crucial to mitigate the risk of abrasion through surface modification and matrix lubrication, whilst fully utilising diamond’s excellent thermal conductivity. Wei et al. [22] increased the thermal conductivity to 112.5 W/(m·K) by adding 47 wt% chromium-coated diamond to the liquid metal; furthermore, the chromium-coated diamond/liquid metal composite achieved a thermal conductivity of 126 ± 6 W/(m·K) at a filler-to-matrix volume ratio of 1:1 [23]. A thermal conductivity of 117.8 ± 1.0 W/(m·K) was achieved by Si et al. [24] using surface-metallized diamond/liquid metal composites with an optimized particle-size distribution, namely a blend of large (400–450 μm) and small (200–250 μm) diamonds. Furthermore, Ki et al. [25] reported that doping with copper nanoparticles (4 vol%) increased the thermal conductivity to 64.8 W/(m·K); Kong et al. [26] found that the LM-W composite slurry doped with tungsten microparticles (40 vol%) achieved a thermal conductivity of 57–62 W/(m·K). Surface metallization is an effective method for improving the wettability at the interface between the filler and the liquid metal. Metal coatings such as chromium can form a carbide transition layer on the diamond surface, thereby enhancing interfacial bonding strength, reducing phonon mismatch, and lowering interfacial thermal resistance [22]. Yet the long-term reliability of liquid metal-based composite thermal interface materials still poses a major challenge to their practical use [18]. Under high-temperature operating conditions, the synergistic effects of interfacial reactions and oxidation can lead to continuous degradation of the material’s microstructure [15,27]. Studies have shown that at temperatures ≤ 150 °C, only the CuGa_2_ IMC forms at the Cu/Ga interface, and its growth follows a parabolic pattern with an activation energy of 14.25 kJ/mol [27]; however, during long-term aging, the growth of CuGa_2_ is accompanied by the formation of microcracks and Kirkendall voids, while oxidation reactions continuously deplete the liquid metal matrix and generate an oxide layer [15,17]. Furthermore, high-temperature and high-humidity environments (85 °C/85% RH) or alternating thermal loads accelerate the failure of the oxide film, further exacerbating performance degradation [15]. Currently, systematic research on the microstructural evolution and performance degradation behavior of liquid metal/copper-coated diamond composite systems during high-temperature aging remains limited, and the failure mechanisms require further elucidation.

Although metal-coated diamond fillers have been widely used to enhance the thermal conductivity of liquid metals, most existing studies have focused on maximising the initial thermal conductivity by optimising filler content, particle size distribution, or coating materials. In contrast, there is relatively little research on the long-term reliability of these composites under high-temperature service conditions. In practical applications, liquid-metal based thermal interface materials are exposed to high-temperature environments over extended periods; interface reactions between gallium and copper (resulting in the formation of the intermetallic compound CuGa_2_) and the oxidation of the liquid-metal matrix act synergistically to degrade the thermal conduction network. At present, systematic studies on the microstructural evolution and performance degradation of LM-CCD composites during high-temperature aging remain limited, and the failure mechanisms are not yet fully understood. Against this backdrop, this paper does not seek to achieve the highest initial thermal conductivity, but rather focuses on the trade-off between thermal performance and long-term reliability.

Based on the above background, this study selected high-purity gallium as the liquid metal matrix and copper-coated diamond (CCD) as the heat-conductivity-enhancing phase. Using a mechanical mixing method, liquid metal/copper-coated diamond (LM-CCD) composite thermal interface materials with different filler contents were prepared. The microstructure and interfacial wetting properties of the materials were characterized using SEM, EDS, contact angle measurements, and other techniques. The study systematically investigated the influence of filler content on the formation of the composite’s thermal conduction network and its thermal conductivity. Combined with finite element numerical simulations using COMSOL Multiphysics 6.2, the study revealed the internal temperature field distribution and heat transfer mechanisms within the composite. Building on this, high-temperature aging tests at 125 °C were conducted to investigate the evolution of the composite’s microstructure and the degradation of its thermal conductivity during aging, and to analyze the synergistic degradation mechanisms of thermal conductivity caused by interfacial reactions and oxidation. Finally, through validation using actual CPU heat dissipation applications, the thermal dissipation advantages of LM-CCD composites compared to commercial thermal grease will be evaluated.

## 2. Materials and Methods

### 2.1. Experimental Materials

The liquid metal matrix used in this study is high-purity gallium (Ga, 99.99% purity, Shenyang Jiabei Co., Ltd., Shenyang, China), with a melting point of 29.76 °C. Diamond micropowder (particle size 150 μm, purity 99.9%) was supplied by Henan Ruixiang Diamond Industry Co., Ltd., Shangqiu, China. CCD (coating thickness approximately 1.9 μm) was purchased from Zhengzhou Nanzuan Industrial Co., Ltd., Zhengzhou, China. Copper sheet (99.9% purity) was purchased from Wenzhou Mingcan Electric Co., Ltd., Wenzhou, China. In addition, the anhydrous ethanol (analytical grade) used in the experiments was purchased from Chengdu Kelong Chemicals Co., Ltd., Chengdu, China and monohydrate citric acid (analytical grade) was purchased from Xilong Science Co., Ltd., Guangzhou, China. Deionized water was prepared using the laboratory’s ultrapure water system.

### 2.2. Preparation of LM-CCD Composite Materials

**Figure 1:**
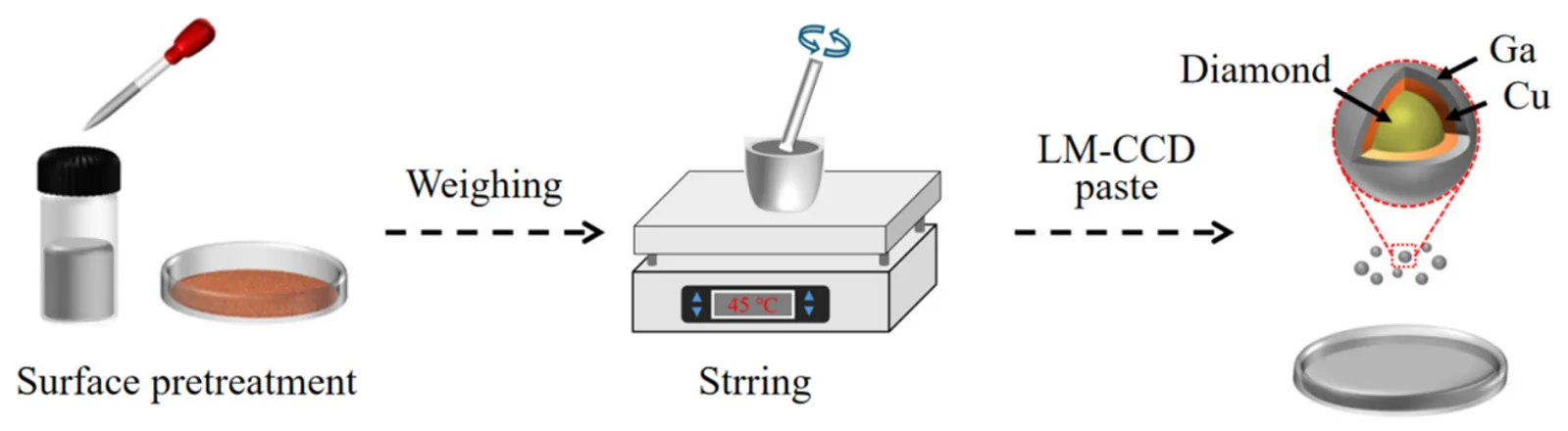
Schematic diagram of the LM-CCD composite fabrication process.

For the removal of any oxides and organic contaminants on the CCD surface, the diamond samples were cleaned in a 10 wt% citric acid solution, successively rinsed with absolute ethanol and deionized water, and then dried at 110 °C under vacuum for 30 min. The LM-CCD composite was prepared using a mechanical mixing method. Dried CCD particles were weighed according to predetermined volume fractions (10 vol%, 20 vol%, 30 vol%, 40 vol%, 50 vol%, and 60 vol%) and placed in a polytetrafluoroethylene crucible together with liquid gallium. To prevent gallium from solidifying during mixing due to temperatures below its melting point, the crucible was placed on a constant-temperature heating block set at 45 °C. Subsequently, a glass rod was used to stir rapidly and continuously in the same direction for approximately 10–15 min until the CCD particles were completely wetted by the gallium and a uniform paste-like mixture was formed, yielding the LM-CCD composite thermal interface material sample. Figure 1 presents a schematic diagram of the fabrication process.

### 2.3. Material Characterization and Performance Testing

A scanning electron microscope (SEM, TESCAN MIRA LMS) (TESCAN, Brno, Czech Republic) was used to observe the surface topography of the raw CCD particles and the composite material; a complementary energy-dispersive X-ray spectrometer (EDS, Oxford Xplore 30) (Oxford Instruments, Oxford, UK) was used to perform area scans and point analyses of the surface composition and distribution of the materials. For particle size analysis, ImageJ 1.54g software was used to perform statistical analysis of the CCD particles in SEM images, measuring the geometric mean particle size of each particle. The results indicate that the particle size of the CCD particles is primarily distributed within the range of 150–180 μm, which is consistent with the nominal particle size provided by the supplier (see Supplementary File, Figures S1 and S2). As particle size was not the focus of this study, a single particle size was used in this paper, and the particle size distribution was employed solely to confirm the uniformity of the CCD raw material used. Wettability testing was performed using the sessile drop method: a drop of high-purity gallium was placed on the surface of a polished copper plate, and a contact angle measuring instrument was used to record the droplet profile and measure the contact angle via software. The test temperature was 45 °C. The thermal conductivity of composite materials was measured using the steady-state heat flow method with a Xiangtan Instruments DRL-IV Thermal Conductivity Tester, in accordance with the ASTM D5470 standard [28]. The composite material was placed between two standard copper blocks (30 mm in diameter, 3 mm thick) and tested under conditions of constant pressure (50 N), a cold-end temperature of 20 °C, and a hot-end temperature of 70 °C. During thermal conductivity testing, the thickness of the sample layer was controlled to 1 mm using nylon shims. Each sample was measured independently five times under identical conditions; the average value was taken as the final result, and error bars were plotted. In the high-temperature aging test, three parallel samples were taken at each aging time point, and each sample was measured five times under identical conditions. The temperature gradient across the upper and lower copper blocks and the corresponding heat flux were measured, from which the equivalent thermal conductivity was derived according to Fourier’s law. The high-temperature aging test was conducted in a constant-temperature oven at 125 °C for 336 h in an air atmosphere. The LM-CCD composite samples were placed in open ceramic crucibles without external clamping pressure during aging, which is a commonly used accelerated aging method to evaluate the intrinsic thermal stability of the material. At predetermined intervals (16, 72, 96, 168, 240, and 336 h), samples were removed, cooled to room temperature, and then subjected to thermal conductivity measurements and surface morphology observations. In practical heat dissipation application verification, the LM-CCD composite material was applied as a thermal interface material between the CPU chip and the heat sink. A K-type thermocouple system (X5-9, KIC, USA) was used to monitor the CPU die temperature and heatsink baseplate temperature in real time. The CPU was stress-tested using AIDA64, and its package power consumption during steady-state operation was approximately 65 W. Tests were conducted under identical ambient temperatures (25 °C) and CPU workloads, with each test run lasting 30 min. Steady-state temperature differences were recorded and compared with those of commercial thermal grease and pure gallium. HUIYA HY2250 commercial silicone-based thermal grease (~5 W/(m·K)) was selected as the reference. The application conditions were identical: the same heat sink spring clips were used and tightened to the same degree, whilst the amount applied was controlled as consistently as possible using a scraper.

### 2.4. Finite Element Simulation

**Figure 2:**
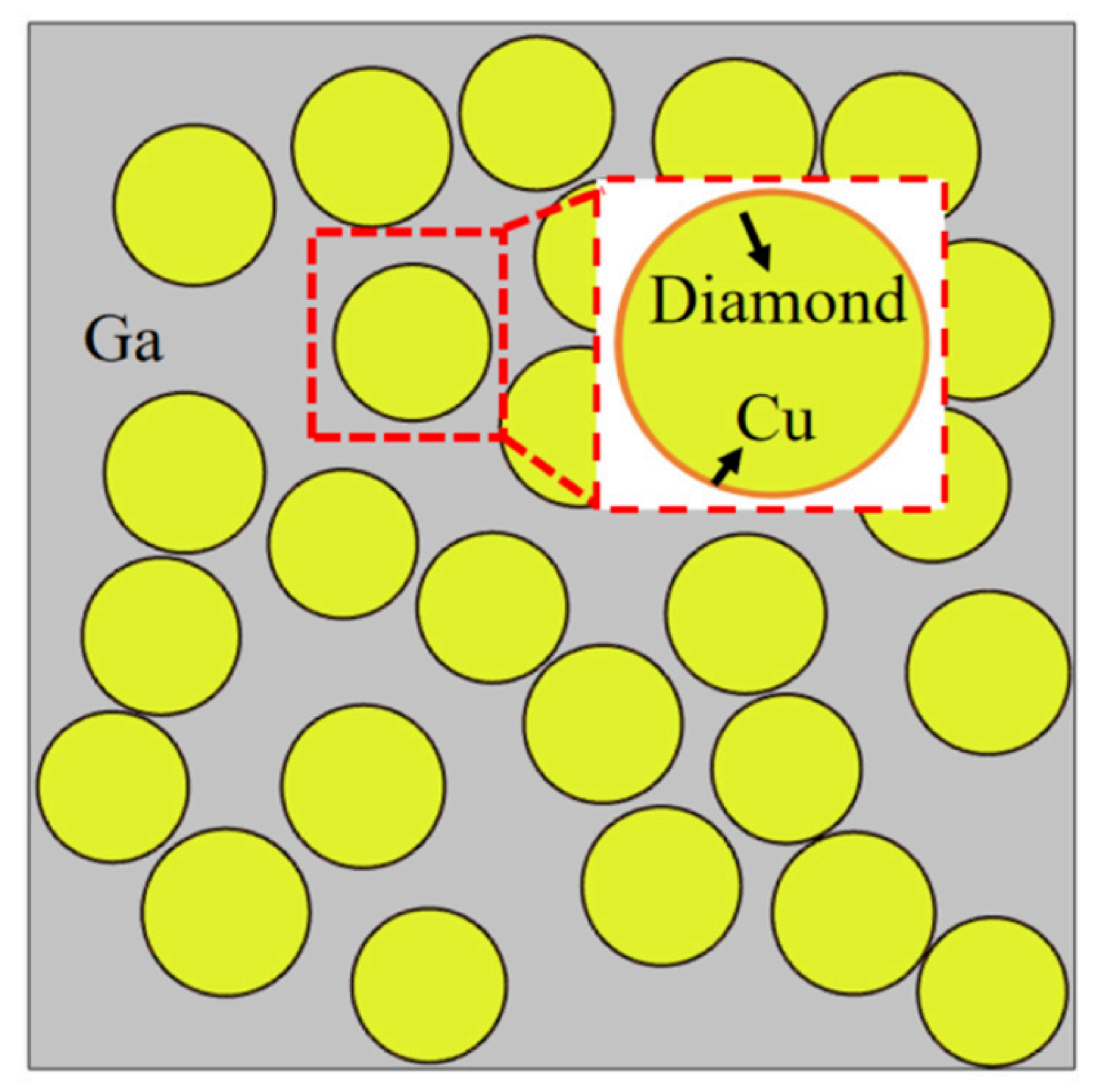
Schematic diagram of a finite element model illustrating the heat transfer mechanism in LM-CCD composite materials.

To thoroughly investigate the effect of CCD particle content on the heat transfer performance of LM-CCD composites, this study established a two-dimensional heat conduction model using COMSOL Multiphysics 6.2 finite element software and conducted numerical simulation analyses. A two-dimensional matrix model measuring 1000 μm × 1000 μm was established, and the positions of the CCD particles (diameter 150 μm) were randomly generated using MATLAB R2024b software and distributed within the liquid metal matrix. A copper coating, in the form of a solid annular domain, enveloped the surface of the diamond particles with a thickness of 1.9 μm (consistent with the calculated actual copper coating thickness). A schematic representation of the finite element model is provided in Figure 2. Corresponding geometric models were established for different filler contents, with the number of particles increasing linearly with the volume fraction. The thermal properties of each material are listed in Table 1. Additionally, to account for the additional contact thermal resistance between the copper coating and liquid gallium caused by phonon mismatch, interfacial defects, and differences in wettability, a thin-layer boundary condition was applied to the outer boundary of the copper coating (i.e., the interface between copper and gallium). This thin layer does not introduce an actual geometric thickness; instead, its equivalent surface thermal resistance was specified. A constant temperature of 343.15 K was applied to the upper boundary of the model, and 293.15 K to the lower boundary, simulating the typical temperature difference experienced by thermal interface materials during actual service. The left and right boundaries were set to insulation conditions, and the model was meshed using an unstructured triangular mesh. Due to differences in the number and spatial distribution of CCD particles at different filler contents, the total number of elements in each model varies accordingly (typical values ranged from 42,070 to 157,846). The maximum element size does not exceed 20 μm, and the average mesh quality for each model was maintained at 0.80 or higher.

Following the solution of the steady-state temperature field and heat-flux density distribution inside the model, the equivalent thermal conductivity of the composite material was derived from Fourier’s law:

(1) $$k_{\mathrm{eff}}=\frac{q\cdot L}{\Delta T}$$

where *q* is the total heat flux density through the model (W/m^2^), *L* is the thickness of the model, and Δ*T* is the temperature difference across the upper and lower boundaries.

## 3. Results and Discussion

### 3.1. Effect of Filler Content on the Microscopic Thermal Conductivity Network and Thermal Conductivity of Composite Materials

**Figure 3:**
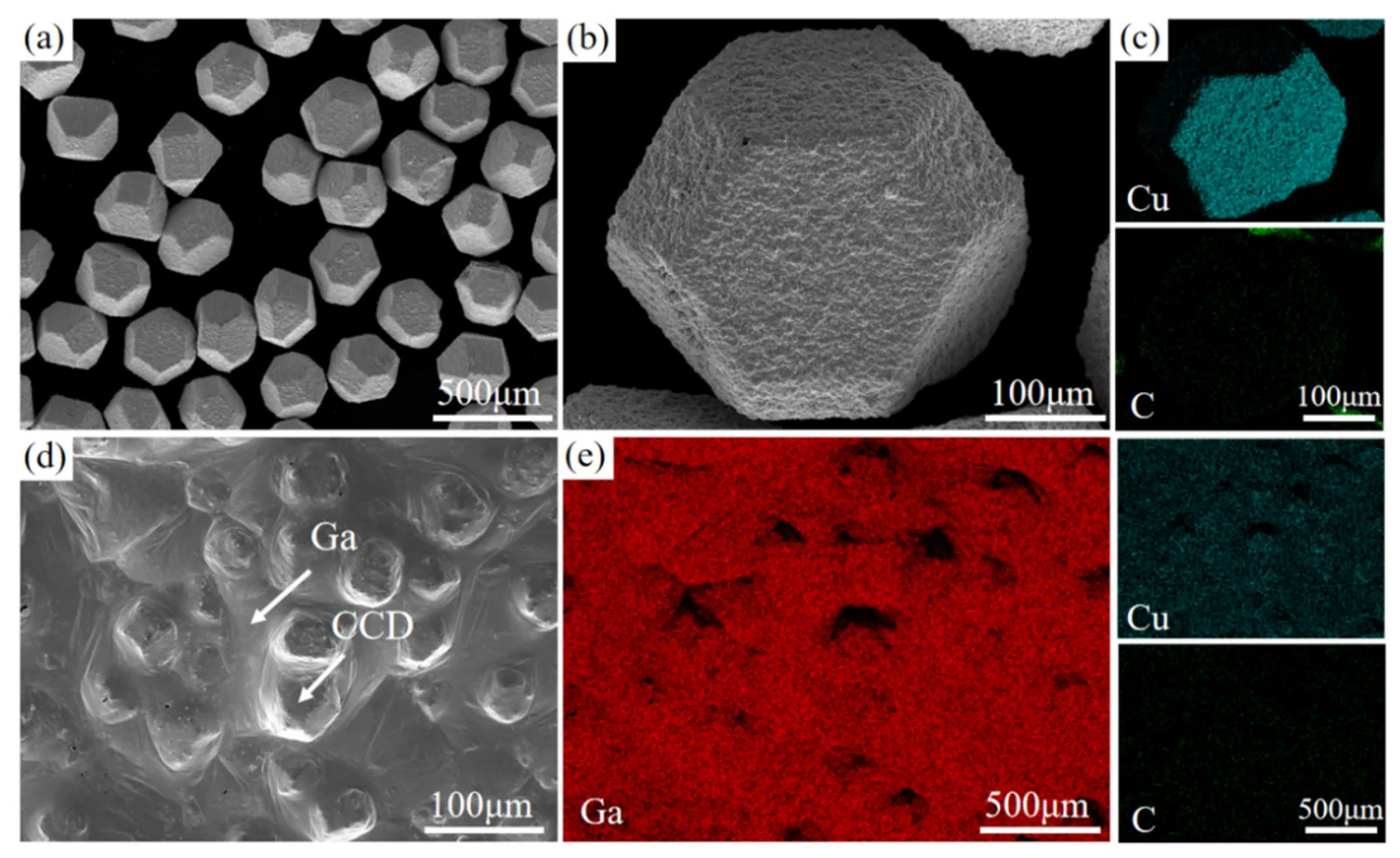
(**a**) Morphology of CCD particles under low-magnification scanning electron microscopy (SEM); (**b**) morphology of CCD particles under high-magnification SEM; (**c**) energy-dispersive X-ray spectroscopy (EDS) analysis corresponding to (**b**); (**d**) SEM image of the LM-CCD composite surface; (**e**) EDS analysis corresponding to (**d**).

Figure 3a,b show the SEM morphologies of diamond particles on the surface of CCD particles after metallization, at high and low magnifications, respectively. The EDS spectral analysis results in Figure 3c indicate that copper is uniformly distributed on the particle surface, with no obvious agglomeration or exposed areas. In the LM-CCD composite (Figure 3d), the CCD particles are encapsulated by a liquid gallium matrix, with no visible pores. The EDS elemental distribution (Figure 3e) shows that gallium is uniformly distributed throughout the composite, demonstrating that gallium exhibits good wettability toward the CCD particles. This improvement can be ascribed to the copper coating, which effectively reduces the interfacial energy between the liquid metal and the reinforcing phase. It also overcomes the poor wettability between diamond and liquid metal, a problem that prevents the liquid metal from spreading effectively over the diamond surface without surface metallization, thereby laying the foundation for a highly efficient thermal conduction network.

**Figure 4:**
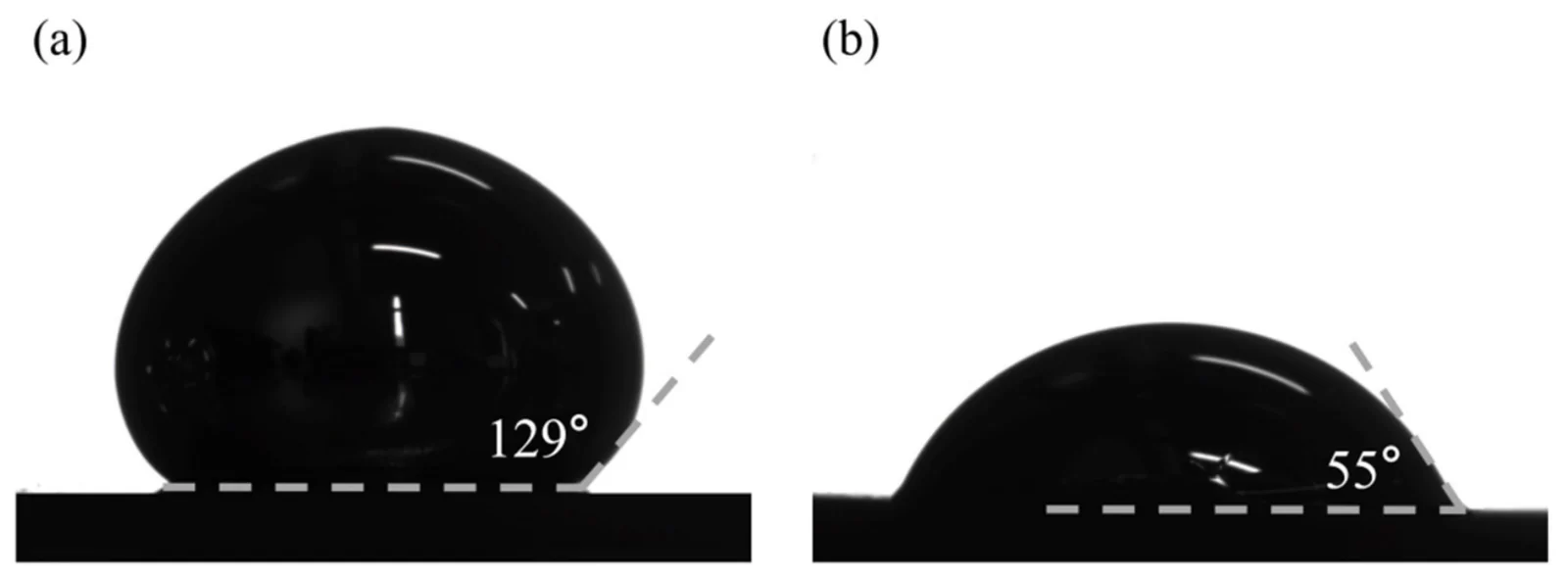
Wetting behavior of liquid gallium on a copper surface: (**a**) wetting behavior of Ga on a copper surface after exposure to air; (**b**) wetting behavior of Ga on a copper surface after removal of the oxide layer by treatment in an HCl atmosphere.

To quantitatively evaluate interfacial wettability, this study measured the contact angle of liquid gallium on a copper surface (Figure 4). The experimental results show that liquid gallium, with its surface oxide layer removed, exhibits a small contact angle (approximately 55°) on the copper surface, demonstrating good wetting properties; however, after exposure to air, a dense Ga_2_O_3_ oxide film forms on the surface of the gallium droplet, causing the contact angle to increase significantly (to approximately 129°) and the wetting properties to deteriorate markedly. This result confirms that the presence of a copper coating effectively promotes interfacial wetting between the liquid metal and the diamond filler; not only does it eliminate the intrinsic non-wetting problem between the diamond surface and the liquid metal, but it also establishes a low-thermal-resistance interfacial bridge for heat transfer, which is consistent with the effects of the chromium/copper dual-layer coating strategy reported in the literature [24]. At the same time, diamond possesses an exceptionally high intrinsic thermal conductivity. However, as it is inherently abrasive, the copper coating acts as a soft buffer layer, effectively mitigating the diamond’s inherent abrasiveness. Furthermore, the liquid gallium matrix exhibits excellent fluidity and lubricity, filling the gaps between the CCD particles under clamping pressure and providing a hydrodynamic lubrication effect. This further reduces the likelihood of localised stress concentration and surface scratching. Consequently, the copper coating and the liquid metal matrix together enable the safe utilisation of diamond’s high thermal conductivity, whilst significantly reducing the risk of abrasion compared to uncoated diamond fillers.

**Figure 5:**
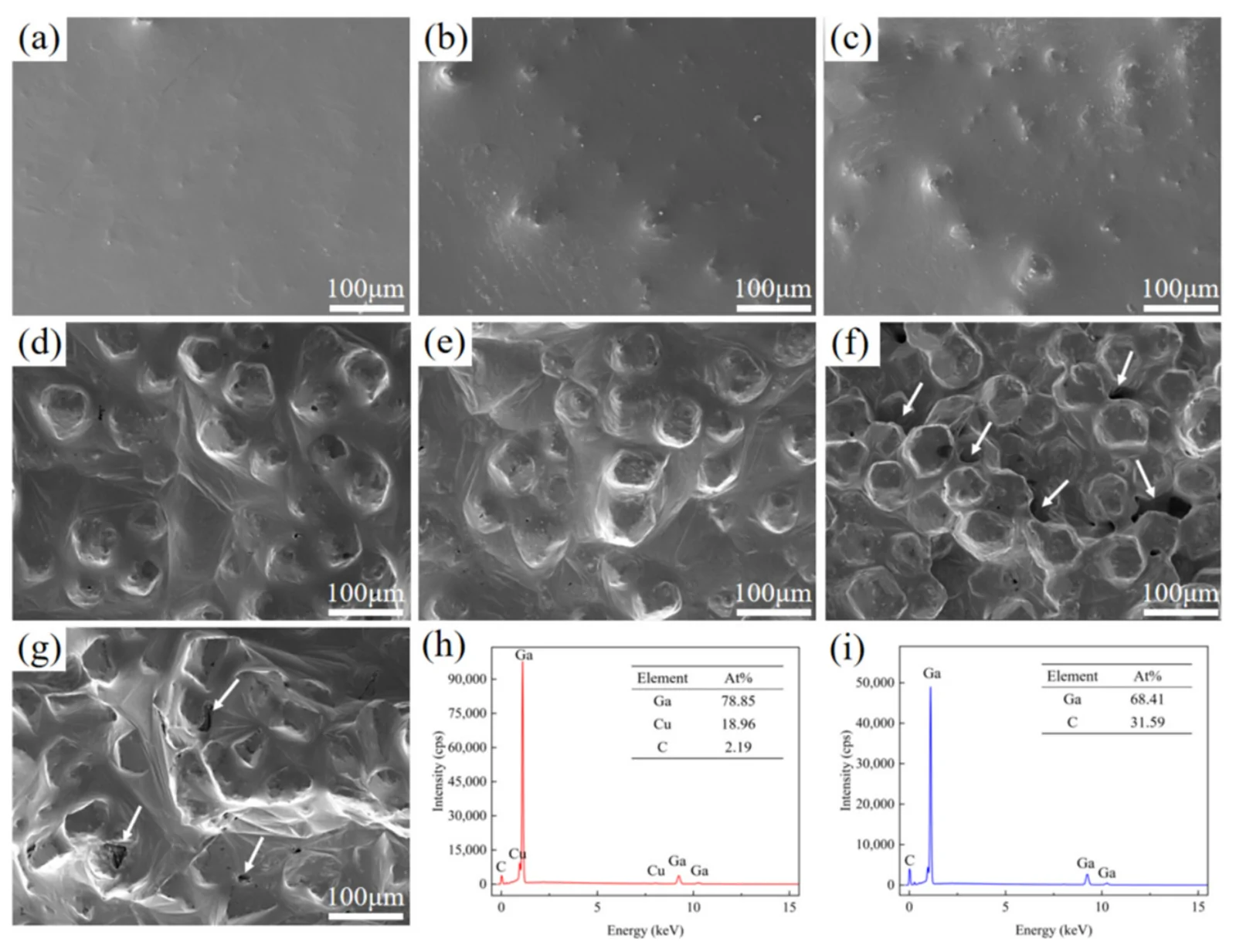
SEM surface morphologies of composites at different filler contents: (**a**) CCD filler content of 10 vol%; (**b**) 20 vol%; (**c**) 30 vol%; (**d**) 40 vol%; (**e**) 50 vol%; (**f**) 60 vol%; (**g**) 50 vol% LM-D composite; (**h**) planar EDS analysis corresponding to (**e**); (**i**) planar EDS analysis corresponding to (**g**).

The volume fraction of the filler has a decisive influence on the formation of the microscopic thermal conduction network within the composite material. Figure 5 shows the SEM surface morphology of LM-CCD composites at different CCD filler contents. When the CCD filler content is below 50 vol% (Figure 5a–d), the particles are completely immersed and uniformly dispersed in the LM matrix. As the filler content gradually increases, the distance between CCD particles gradually decreases, and some particles begin to come into contact with one another, forming localized thermal conduction chains. When the filler content reaches 50 vol% (Figure 5e), the CCD particles form a relatively complete, interpenetrating thermal conduction network within the liquid metal matrix. Effective face-to-face or point-to-point contact between particles is achieved through the copper coating, providing continuous, low-thermal-resistance phonon transport pathways for heat transfer. By comparison, at the same filler content (50 vol%), this study further examined the microstructure of the liquid metal/diamond (LM-D) composites (Figure 5g). It can be seen that some diamond particles were not completely encapsulated by LM; due to the poor intrinsic wettability between the unmetallized diamond and LM, the LM could not spread effectively. Even when physical contact occurs between particles at high filler contents, such interfacial gaps still introduce significant phonon scattering and interfacial thermal resistance, severely compromising the actual heat transfer efficiency of the thermal network. When the CCD filler content is further increased to 60% (Figure 5f), the microstructure of the composite deteriorates significantly, with distinct voids and pore defects becoming observable. At this stage, the CCD particles transition from a state of complete immersion to a liquid-bridge configuration within the LM. This type of connectivity results in inadequate filling of the interparticle voids. The presence of these voids not only disrupts the continuity of the thermal conduction network but also introduces air gaps, severely hindering the effective transfer of heat. Furthermore, as the CCD particles used in this study have a narrow particle size distribution and the filler content varies systematically within the range of 10–60 vol%, the changes in thermal conductivity are primarily attributable to the filler content and the formation of the heat conduction network; the effects of variations in particle size are not considered at this stage. At the same time, the particle size of the CCD filler also influences the thermal conductivity of the composite material by altering the interfacial area, particle packing and network continuity. Future work will involve a more systematic investigation into the effects of CCD particle size and particle size distribution on the thermal conductivity of LM-CCD composites.

**Figure 6:**
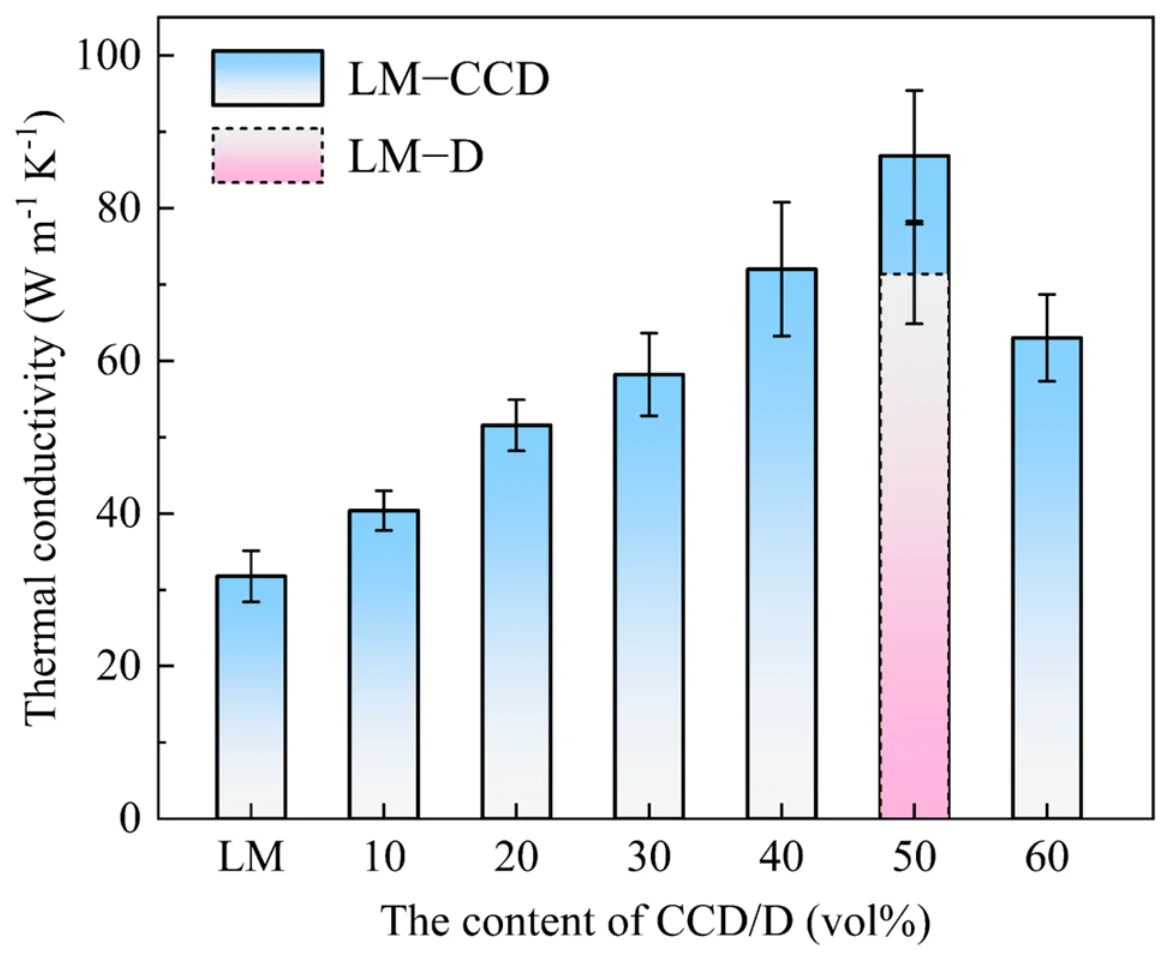
Relationship between the thermal conductivity of LM-CCD/LM-D composites and filler content.

Figure 6 illustrates the variation of thermal conductivity of the LM-CCD composite TIM as a function of the CCD filler volume fraction. With the CCD content progressively increasing from 10 vol% to 50 vol%, the thermal conductivity of the composite continuously increases, reaching a peak value of about 86.9 W/(m·K) at 50 vol%. This trend is highly consistent with the microstructural observations in Figure 5. To verify the necessity of the copper coating for enhancing thermal conductivity, we also prepared and tested LM-D composites with different filler contents (10–60 vol%). The average thermal conductivity of LM-D was lower than that of LM-CCD at the same filler content (see Supplementary File, Figure S3). At 50 vol%, the average thermal conductivity of LM-CCD (86.9 W/(m·K)) is higher than that of LM-D (73.5 W/(m·K)) (see Figure 6). It is worth noting that the thermal conductivity of the 40 vol% LM-CCD composite is close to that of the 50 vol% LM-D composite, within the measurement error range. This suggests that when the diamond surface is coated with copper, a similar thermal conductivity can be achieved at a lower filler content. More importantly, increasing the filler content of the LM-D composite to 50 vol% would result in poorer wettability and more interfacial defects, as shown by the microstructure in Figure 5g. Therefore, the copper coating is essential as it enhances interfacial wettability and network continuity, thereby enabling efficient heat conduction at lower filler contents. As a highly thermally conductive reinforcing phase, an increase in the volume fraction of CCD implies a higher proportion of highly thermally conductive components in the composite; at the same time, the copper coating acts as an interfacial transition layer, effectively reducing the interfacial thermal resistance between the diamond and the liquid metal, allowing phonons to cross the interface more efficiently. When the filler content was further increased to 60 vol%, the thermal conductivity showed a marked decrease. This “bell-shaped” trend is consistent with the patterns reported in the literature regarding the variation of thermal conductivity in liquid metal/diamond composites with filler content [23]. Excessively high filler content leads to the formation of voids and defects within the composite, compromising the integrity of the thermal conduction network. Furthermore, an excess of solid-phase particles increases the viscosity of the composite, making it difficult for the liquid metal to fully wet and fill the narrow gaps formed by the aggregation of CCD particles, which further degrades the efficiency of interfacial heat transfer.

**Figure 7:**
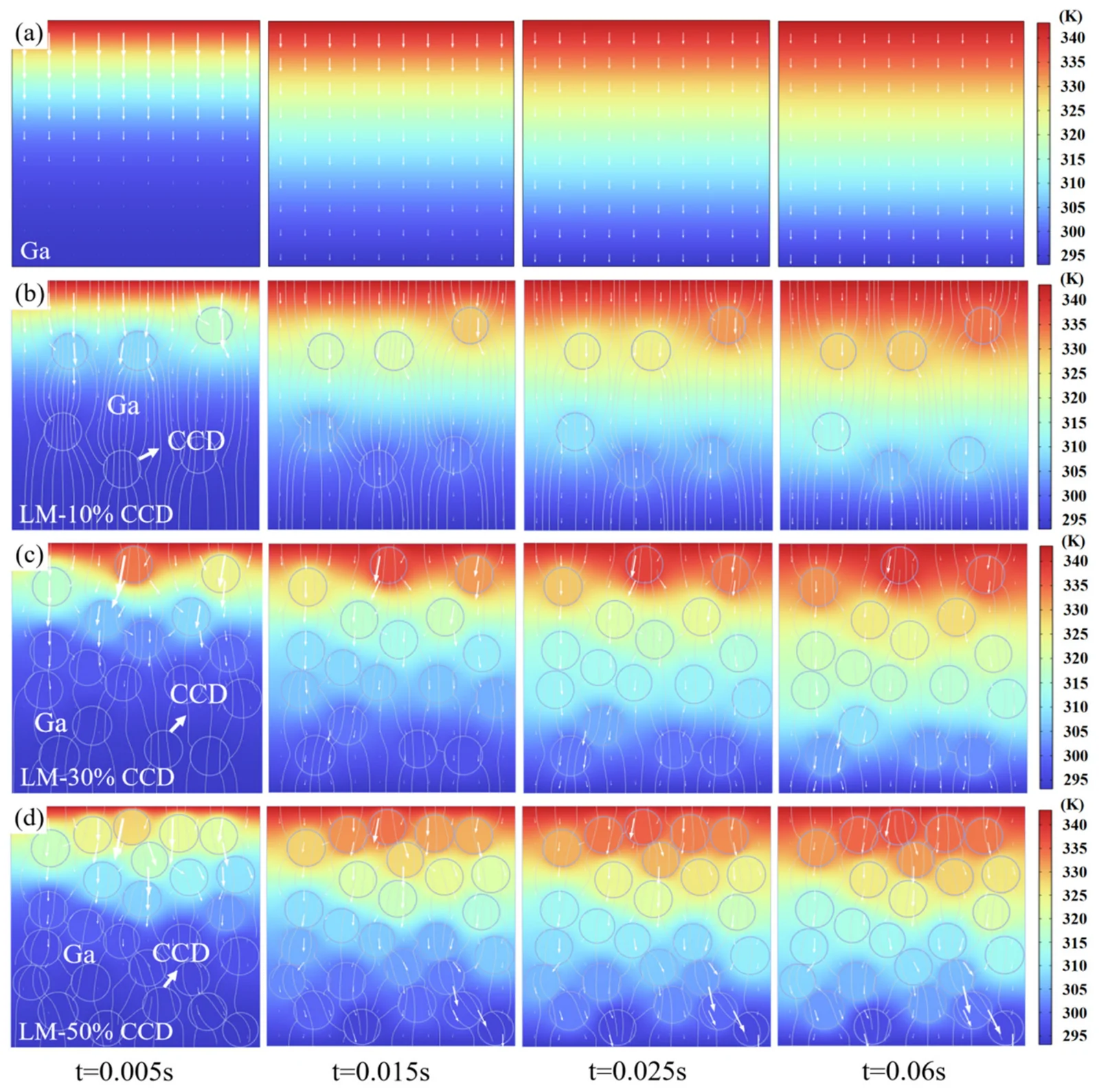
Contour plots showing the steady-state temperature field distribution of LM-CCD composite materials at different CCD contents: (**a**) Ga; (**b**) 10 vol% CCD filler; (**c**) 30 vol%; (**d**) 50 vol%.

**Figure 8:**
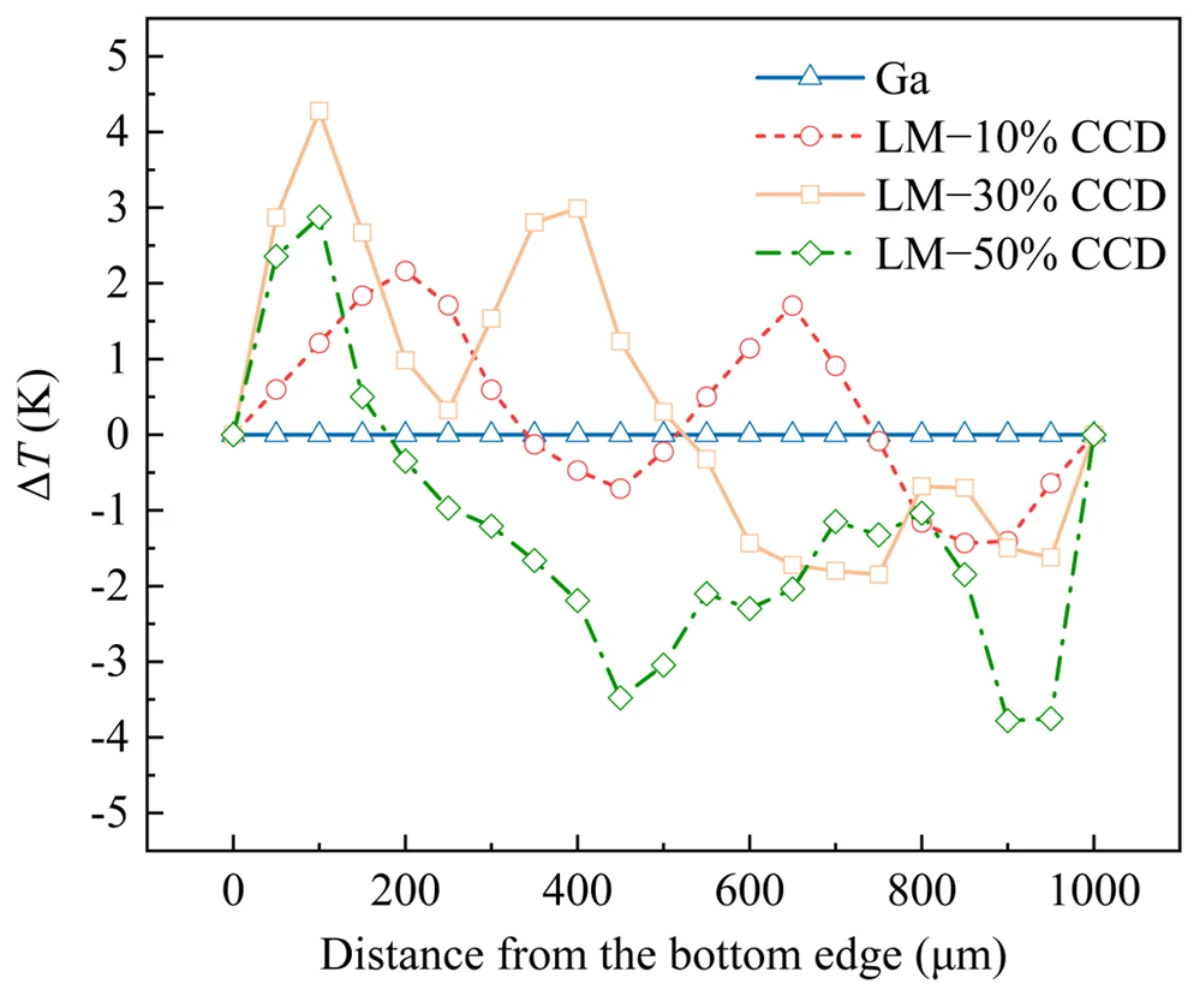
Temperature difference distribution curves for composite materials relative to a pure gallium reference at different CCD contents.

**Figure 9:**
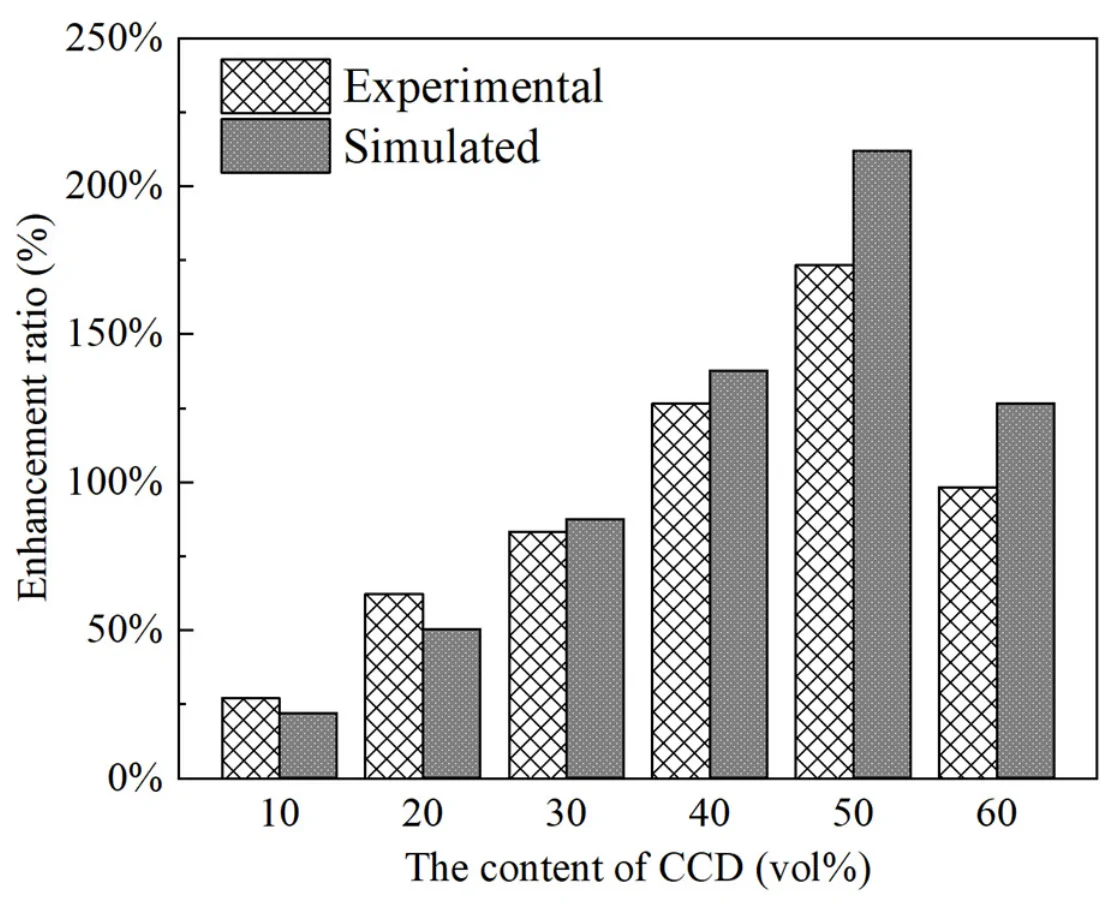
Comparison of experimental and simulation results for the increase in thermal conductivity of composite materials (The simulated values were calculated by the COMSOL finite element model described in Section 2.4).

This study further investigated the heat conduction mechanism of LM-CCD composites through finite element simulation. Figure 7 shows the time-dependent temperature distribution within the composite at different CCD particle contents. As can be observed from the figure, heat is primarily transferred along paths formed by the highly thermally conductive CCD particles within the composite; the CCD particles effectively guide the directional transfer of heat. To quantitatively evaluate the effect of different CCD contents on thermal conductivity enhancement, this study selected four characteristic straight lines along the heat flux direction (y-direction) at distances of 200 μm, 400 μm, 600 μm, and 800 μm from the *y*-axis. Temperature data were collected at 50 μm intervals along each line, and the average temperature at the same position across the different lines was calculated. Subsequently, using the average temperature of the pure liquid gallium model as the baseline (Δ*T* = 0), the temperature differences relative to the pure gallium baseline at each sampling location were calculated for different CCD contents. The results are shown in Figure 8. Compared to the low-content group, the Δ*T* curve for the high-content group exhibited negative values over more than 80% of the path, with a negative deviation reaching −4 K at its peak. This indicates that high diamond content forms a long-range, continuous thermal conduction framework within the matrix. This structure effectively reduces phonon scattering at interfaces, providing a low-thermal-resistance pathway for heat transfer throughout the composite material and effectively reducing heat accumulation along the entire path. Furthermore, both experimental and simulation results show that the increase in thermal conductivity follows a trend of first rising and then falling with increasing CCD content (as shown in Figure 9), reaching a maximum at 50 vol%. The simulation data shown in Figure 9 are derived from the COMSOL finite element model described in Section 2.4, which includes randomly distributed CCD particles, a copper coating and the equivalent interfacial thermal resistance at the Cu/Ga interface. The two sets of results agree well, validating the accuracy of the model.

### 3.2. Microstructural Evolution and Performance Degradation During High-Temperature Aging

**Figure 10:**
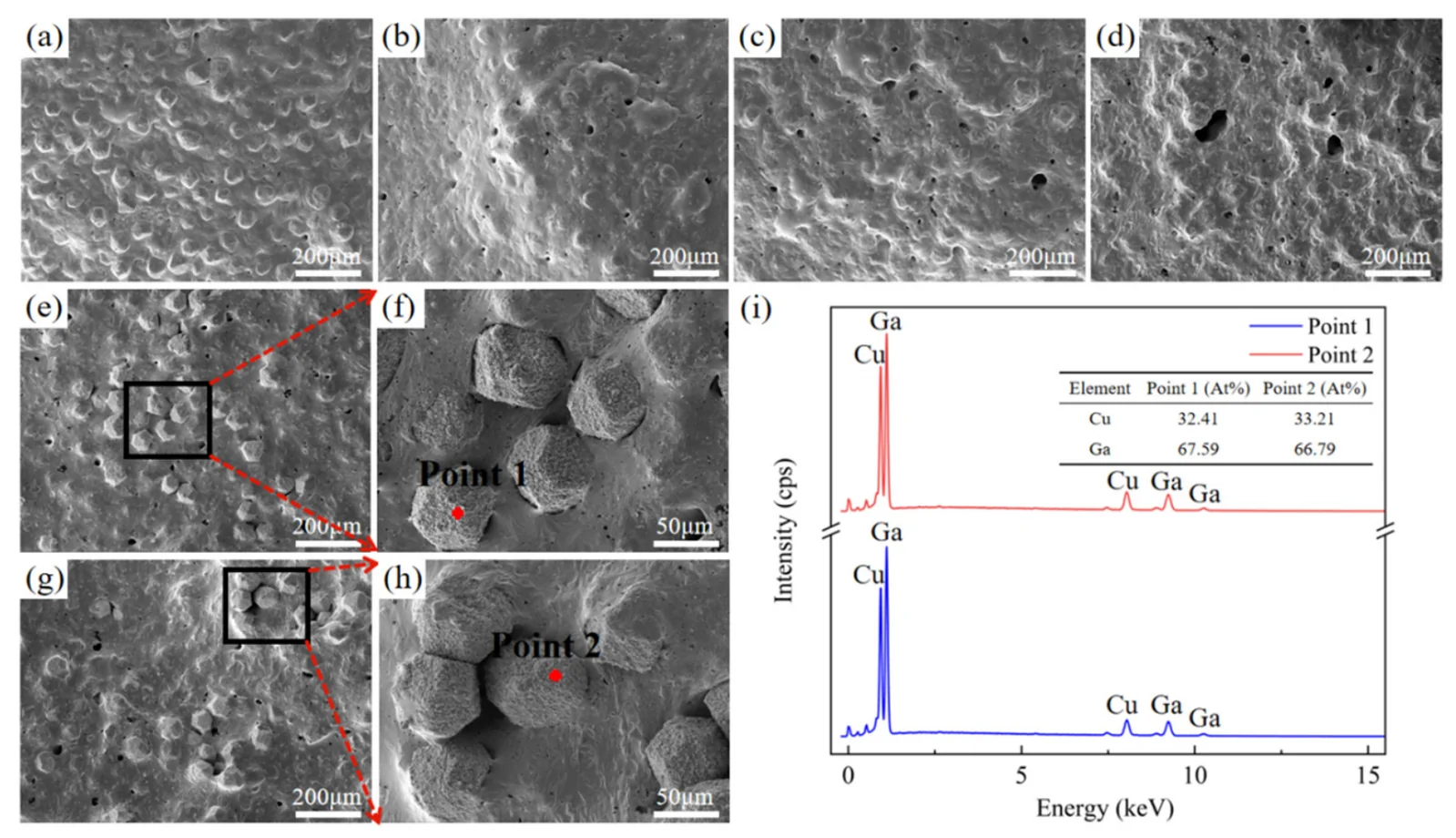
Surface conditions of the 50 vol% LM-CCD composites at different aging times: (**a**) 16 h; (**b**) 72 h; (**c**) 96 h; (**d**) 168 h; (**e**) 240 h; (**f**) enlarged view of the rectangular area in (**e**); (**g**) 336 h; (**h**) enlarged view of the rectangular area in (**g**); (**i**) EDS results for points 1 and 2.

Thermal interface materials are subjected to high-temperature operating environments over long periods during actual service, and the stability of their microstructure directly determines the long-term reliability of the device. The LM-CCD composite with 50 vol% CCD, which exhibited the highest initial thermal conductivity, was selected for the high-temperature aging tests. Figure 10 shows SEM images of the surface morphology of the LM-CCD composite after aging for different durations at 125 °C. In the early stages of aging (16 h, Figure 10a), the composite surface was generally smooth, with CCD particles uniformly embedded in the liquid metal matrix. The interfaces between the particles and the matrix were distinct and well-bonded, with no obvious voids or cracks observed, indicating that the composite in its original state possessed a dense microstructure. As the aging time increased, voids gradually began to appear on the composite surface, and material defects became increasingly evident with prolonged aging (Figure 10b–d). When the aging time reached 240 h (Figure 10e), the surface morphology of the composite deteriorated significantly, accompanied by a small amount of granular precipitates distributed across the matrix surface. According to EDS analysis (Figure 10i), the atomic ratio of Cu to Ga in the precipitates was close to 1:2; EDS analyses were also carried out at several locations in Figure 10f (see Supplementary File, Figures S4 and S5). According to the EDS results, the Cu/Ga atomic ratio in the crystals ranged from 0.45 to 0.65 (see Supplementary File, Table S1), which can be preliminarily identified as CuGa_2_. CuGa_2_ possesses a tetragonal crystal structure. To further verify the CuGa_2_ phase detected by EDS, XRD analysis was performed on the LM-CCD composite material after aging at 125 °C for 240 h; the detailed XRD patterns are shown in Supplementary File Figure S6. The characteristic diffraction peaks in the XRD pattern matched the standard card for CuGa_2_ (PDF#04-003-1650) well, consistent with the EDS analysis and the observed microstructural degradation. This further confirmed the chemical composition of the material on the surfaces of the exposed particles.

**Figure 11:**
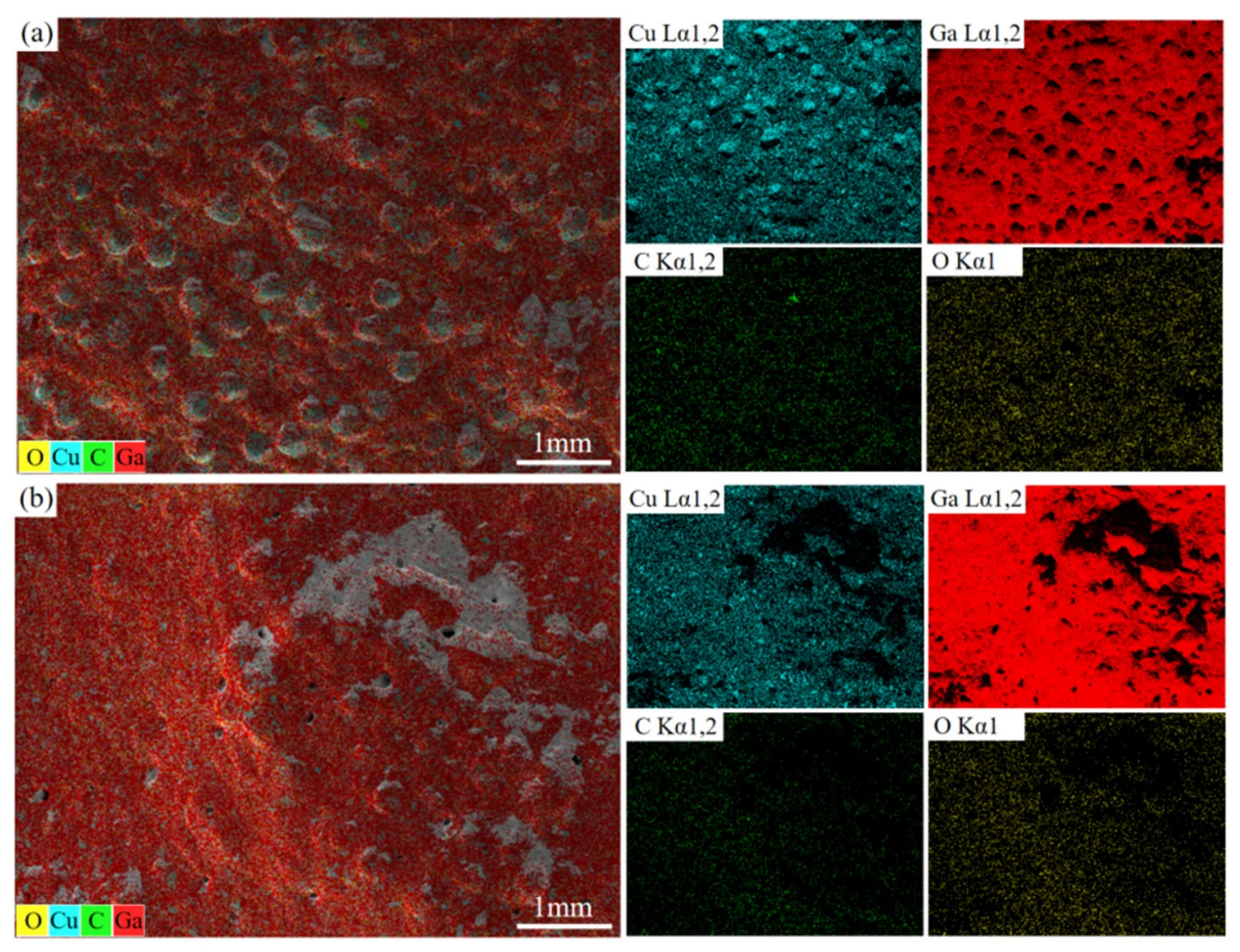

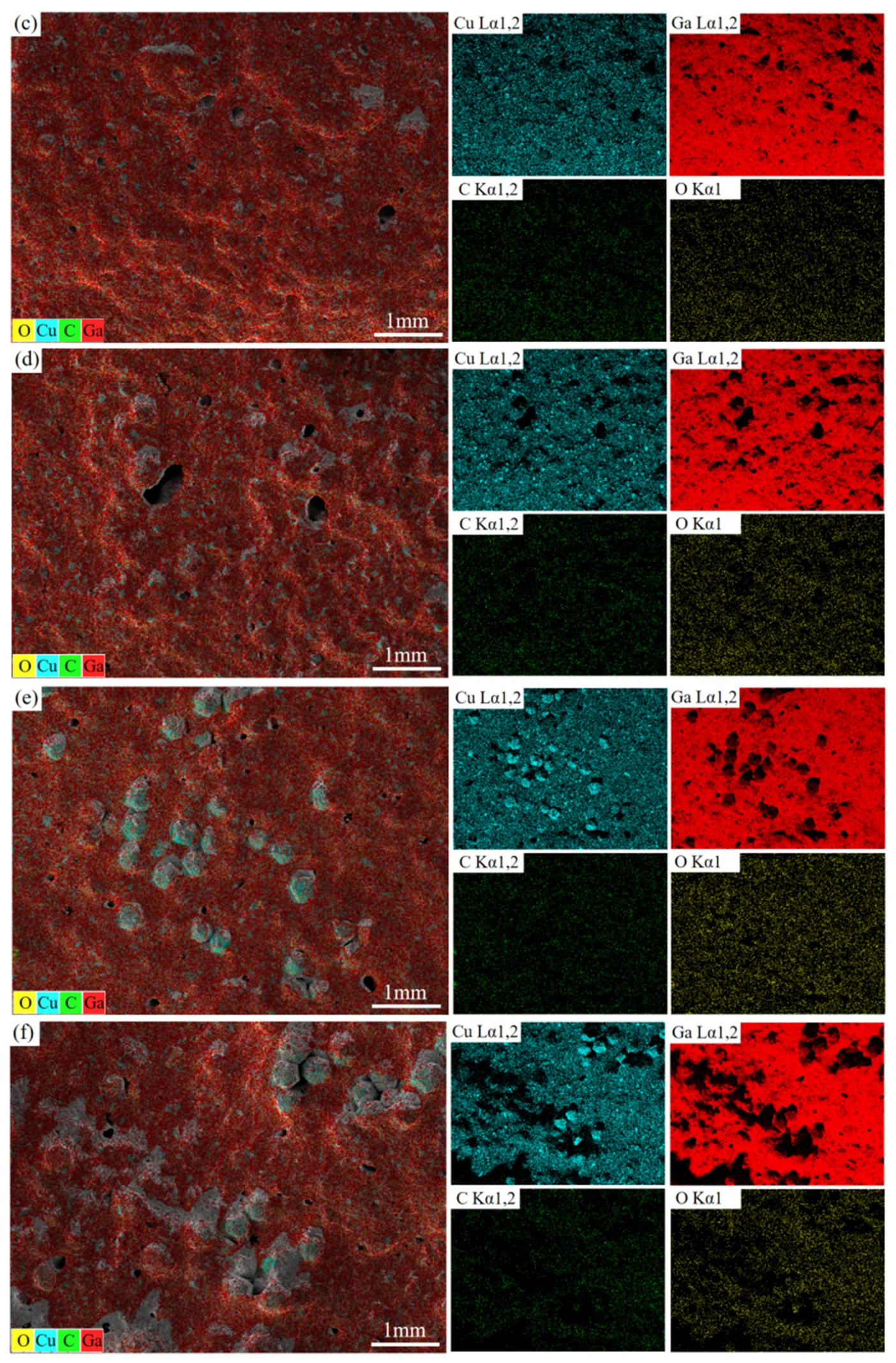
EDS surface scan results of the 50 vol% LM-CCD composite at different aging times shown in Figure 10: (**a**) 16 h; (**b**) 72 h; (**c**) 96 h; (**d**) 168 h; (**e**) 240 h; (**f**) 336 h.

Figure 11 shows the EDS surface scan results of the aged sample surface shown in Figure 10 and provides a systematic analysis of the distribution of Ga, Cu, C, and O elements. The EDS mapping results show that, as the aging time increased, the oxygen (O) signal on the material surface gradually intensified, indicating that oxidation reactions occurred in the composite material under high-temperature air conditions. At the same time, the reaction between gallium and copper produced the IMC CuGa_2_, and these reaction products aggregated at the interface and gradually grew, ultimately leading to defects in the material.

**Figure 12:**
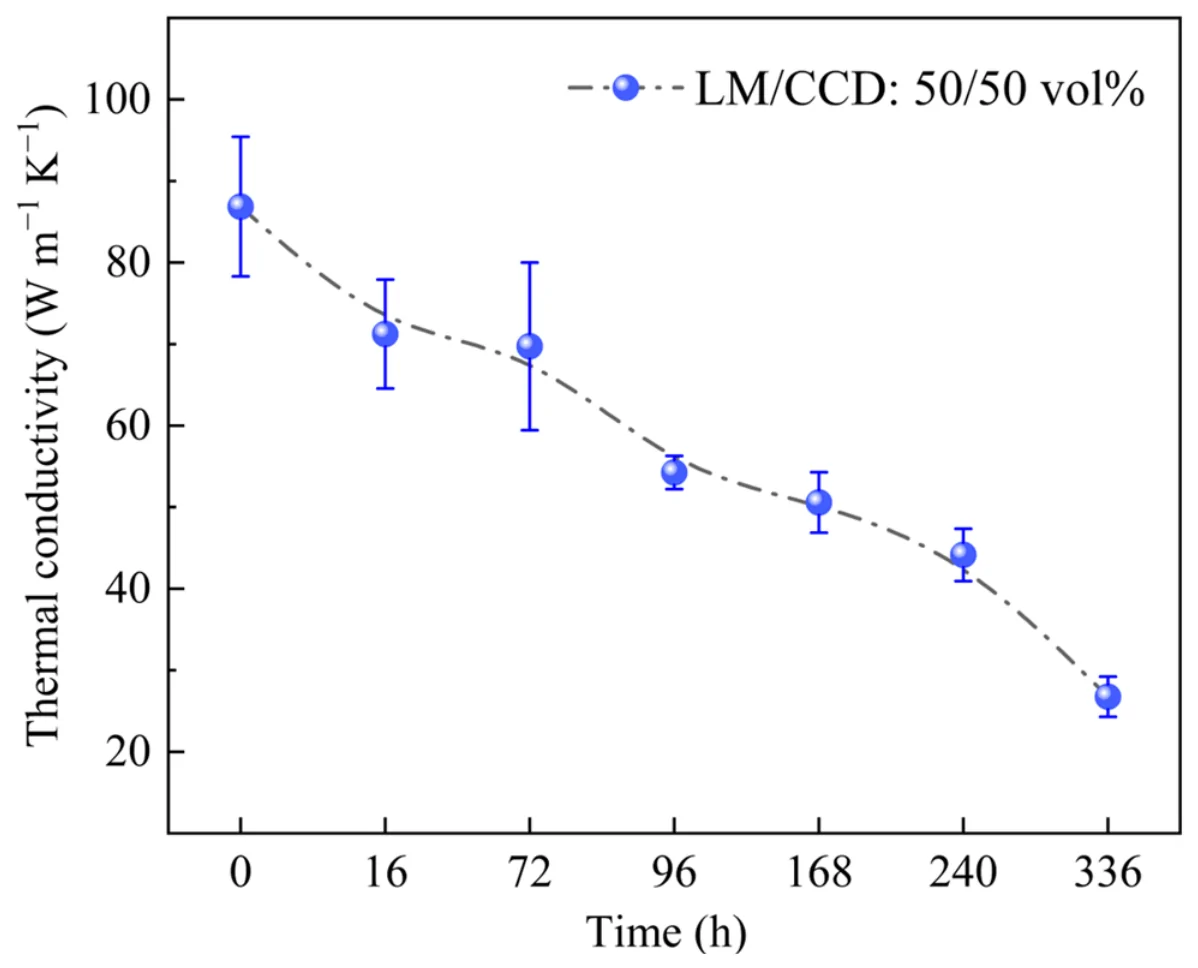
Curve showing the change in thermal conductivity of LM-CCD composite material as a function of aging time following high-temperature aging.

Figure 12 shows the curve of the thermal conductivity of the LM-CCD composite as a function of aging time following high-temperature aging. After 72 h of aging, the thermal conductivity dropped by 19.4% (from 86.9 to 70.0 W/(m·K)), indicating a relatively moderate initial degradation. Based on observations of the microstructure before aging (Figure 5e) and during the early stages of aging (Figure 10a–b), only minor surface defects were present, and no significant damage to the thermal conduction network had yet occurred. As the aging time extended to 240 h, the thermal conductivity exhibited an accelerated decline, further decreasing to 44.1 W/(m·K), representing a total decrease of 49.3% relative to the initial value. After 336 h of aging at 125 °C, the thermal conductivity of the 50 vol% LM-CCD composite fell to approximately 27 W/(m·K), approaching that of pure gallium. This was due to the continuous growth of the interfacial reaction product, the CuGa_2_ IMC, which was accompanied by a volume expansion effect that introduced localized stress at the interface; simultaneously, oxidation reactions (as indicated by the enhanced O signal in Figure 11) continuously consumed the liquid metal matrix and formed an oxide layer. The synergistic effect of these two processes leads to the formation and propagation of microcracks and pores within the composite, disrupting the original continuous heat conduction pathways. While the formation of a moderate amount of CuGa_2_ has a limited impact on interfacial heat transfer, the volumetric expansion and stress concentration caused by excessive growth degrade the heat conduction pathways [27], obstructing phonon transport and causing a sharp increase in interfacial thermal resistance. Under such accelerated aging conditions, the thermal enhancement effect of the CCD thermal network is almost entirely lost. This aging test was conducted in air and without sustained clamping pressure, which may accelerate oxidation and interfacial reactions more readily than under actual CPU operating conditions. In actual CPU packages, thermal interface materials are typically clamped between the processor lid and the heat sink, which can partially suppress oxidation and leakage, and the operating temperature is usually below 125 °C. Nevertheless, these results clearly demonstrate that suppressing interfacial reactions between Ga and Cu and preventing oxidation are crucial for maintaining the long-term reliability of LM-CCD composites.

The above results indicate that the synergistic effect of interfacial reactions and oxidation during high-temperature aging is the fundamental cause of the degradation in the thermal conductivity of LM-CCD composites; the long-term service reliability of these composites still needs to be improved through interfacial modification or other protective measures. It should be noted that the high-temperature aging tests in this study were conducted in air without sustained clamping pressure; this does not fully represent the actual operating environment of a CPU. Therefore, the reliability results can be regarded as an accelerated assessment of the intrinsic thermal stability of the LM-CCD composite material; further reliability studies under actual packaging conditions are required in the future. At the same time, the copper coating improves the interfacial wettability between diamond and liquid gallium, facilitating the formation of a continuous thermal conduction network. However, during aging, the copper coating also participates in interfacial reactions, forming CuGa_2_, which leads to the degradation of the thermal conduction network. This trade-off between initial thermal performance and long-term reliability should be addressed in future work through interfacial engineering.

### 3.3. Validation of Heat Dissipation Applications of Composite Materials in Actual Electronic Devices

**Figure 13:**
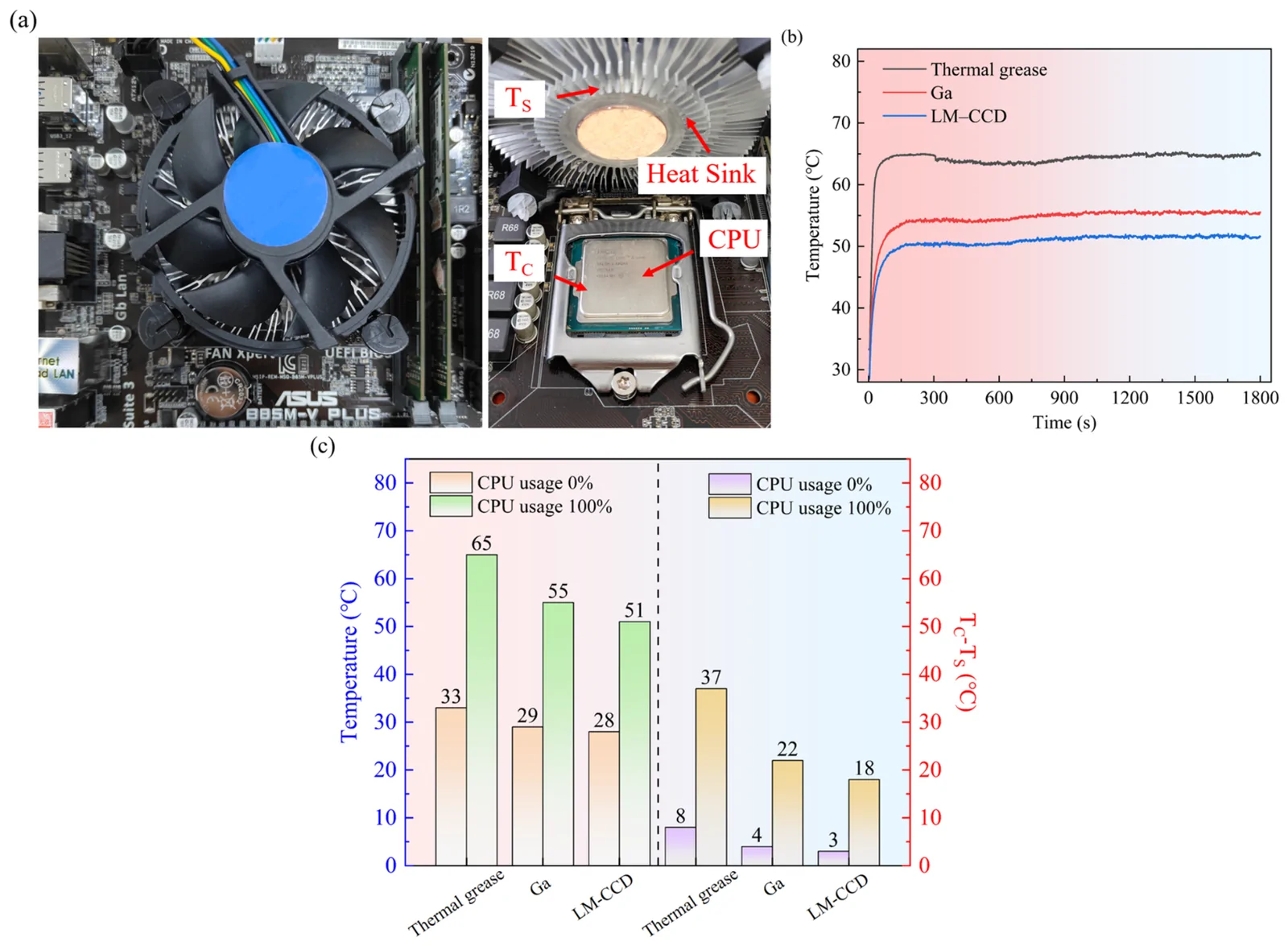
Practical application validation: (**a**) schematic diagram of the cooling system; (**b**) CPU temperature versus time curve; (**c**) comparison of steady-state temperature differences between the CPU and the heat sink.

To assess the real-world thermal dissipation effectiveness of the LM-CCD composite as a TIM, it was utilized at the CPU-heat sink interface and contrasted with commercial thermal paste (HY2250) and pure gallium in the present work. The materials were applied under the same pressure and coating conditions. This comparison aims to verify the practical engineering potential of LM-CCD as a replacement for conventional thermal interface materials. Figure 13a shows a schematic diagram of the heat dissipation verification setup (the test platform utilises an Intel LGA1150 socket, with an Intel Core i5-4690 processor featuring a base TDP of 84 W; the cooler is Intel’s standard copper-base fan): the CPU chip is mounted on top of the test motherboard, and the LM-CCD composite material is applied as the thermal interface material between the CPU and the heat sink base. For a comparison of aluminum-base heat sinks, see Figure S7 in the Supplementary File. The CPU case temperature (*T_C_*) and heat sink temperature (*T_S_*) are monitored in real time using a KIC X5-9 thermocouple, and the temperature difference between the two, Δ*T = T_C_ − T_S_*, is calculated. Figure 13b shows the curve of CPU temperature over time. After running for 30 min under the same workload conditions, the steady-state operating temperature of the CPU using the LM-CCD composite as the thermal interface material was lower than that of the control group using commercial thermal grease. This difference is particularly evident under sustained high-power operating conditions—the CPU temperature in the control group exhibits a continuous upward trend and converges to a higher steady-state value, whereas the steady-state temperature of the CPU using LM-CCD is approximately 14 °C lower than that of the thermal grease and about 4 °C lower than pure gallium. These results indicate that the LM-CCD composite material enables more efficient heat transfer than traditional thermal grease, transferring heat generated by the CPU more rapidly to the heat sink and dissipating it into the environment.

Figure 13c shows a comparison of the steady-state temperature difference between the CPU and the heat sink. The magnitude of the temperature difference, Δ*T*, directly reflects the temperature drop across the thermal interface material; a smaller Δ*T* indicates higher heat transfer efficiency of the thermal interface material. The results show that when using commercial thermal grease, the Δ*T* between the CPU and the heat sink is relatively large (approximately 37 °C); however, when using the LM-CCD composite material, the temperature difference is significantly reduced. The high thermal conductivity and good fluidity of the liquid metal matrix enable it to effectively fill the microscopic irregularities between the CPU top cover and the heat sink, while the introduction of CCD filler further enhances the composite material’s overall equivalent thermal conductivity, establishing an efficient heat transfer pathway from the heat source to the heat sink.

Based on the results of the above practical application tests, the LM-CCD composite material demonstrated superior thermal performance compared to commercial thermal grease in CPU cooling applications, indicating that it holds promising prospects for use as a high-performance thermal interface material in the field of thermal management for high-power electronic devices. It must be acknowledged that this study has primarily focused on the microstructural evolution and high-temperature reliability of LM-CCD composite materials. Future work will involve conducting a comprehensive comparative study of liquid-metal-based thermal interface materials under conditions where the thickness of the interface layer and the interfacial pressure are precisely controlled.

## 4. Conclusions

In this study, LM-CCD composite thermal interface materials with different filler contents were prepared, and their microstructure, thermal conductivity, and high-temperature service reliability were systematically investigated. The following main conclusions were drawn:

(1) Filler content has a decisive influence on the formation of the internal thermal conduction network and the thermal conductivity of the composite material. As the volume fraction of CCD increased from 10 vol% to 50 vol%, the particles gradually transitioned from isolated, dispersed particles to a continuous, mutually contacting thermal conduction framework. At 50 vol%, a complete interconnecting network formed, and the thermal conductivity reached a peak of 86.9 W/(m·K), approximately three times that of pure gallium. Further increasing the filler content to 60 vol% resulted in a significant decrease in thermal conductivity due to the formation of pores and defects. COMSOL finite element simulations verified that the long-range thermal conduction pathways formed by high CCD content can effectively reduce heat accumulation within the composite, providing a theoretical basis for optimizing the filler ratio.

(2) High-temperature aging experiments revealed the performance degradation mechanism of LM-CCD composites during long-term service. After 240 h of aging at 125 °C, the material’s thermal conductivity decreased by approximately 49.3%. After 336 h of aging, the thermal conductivity further decreased to approximately 27 W/(m·K), indicating that the enhancing effect of the CCD network was completely lost under these accelerated aging conditions. The fundamental cause of this performance degradation lies in the formation and aggregation of the interfacial reaction product—the IMC CuGa_2_—as well as the oxidation of the liquid metal matrix. The synergistic effect of these two factors leads to the formation and propagation of microcracks and pores at the interface, disrupting the continuity of the heat conduction pathways. These results indicate that suppressing interfacial reactions and oxidation is a key strategy for enhancing the long-term reliability of liquid metal based TIMs.

(3) Practical CPU cooling validation demonstrated that when a 50 vol% LM-CCD composite was used as the thermal interface material, the chip’s steady-state temperature was reduced by approximately 14 °C compared to commercial thermal grease (~5 W/(m·K)), and about 4 °C lower than pure gallium, showcasing excellent practical heat dissipation capabilities and promising application prospects in the field of thermal management for high-power electronic devices.

In summary, LM-CCD composites offer significant advantages in thermal conductivity and short-term heat dissipation performance; however, their long-term reliability under high-temperature conditions still requires further optimization through strategies such as interfacial modification, oxidation-resistant encapsulation, or alloying control. This study primarily focused on CCD fillers with a single particle size and aging at a constant temperature of 125 °C. Future research could further investigate the effects of particle size distribution optimization, aging at multiple temperatures, and interface modification on long-term reliability. This study provides systematic experimental evidence and theoretical references for the multi-objective design and reliability enhancement of liquid metal-based composite thermal interface materials.

## Figures and Tables

**Table 1:** Finite element simulation parameters.

Materials	Density (kg/m^3^)	Heat Capacity (J/(kg·K))	Thermal Conductivity (W/(m·K))
Copper	8960 [29]	385 [29]	400 [29]
Gallium	6093 [30]	381.5 [30]	28 [30]
Diamond	3515 [31]	520 [31]	2200 [31]

## Data Availability

The authors confirm that the data supporting the findings of this study are available within the article.

## Supplementary Materials

The following supporting information can be downloaded at https://www.mdpi.com/article/10.3390/nano16211340/s1. Figure S1: (a) Raw SEM morphology of CCD particles; (b) schematic diagram showing particle contour extraction and particle size measurement using ImageJ software; Figure S2: Particle-by-particle scatter plot of the geometric mean particle size of CCD particles; Figure S3. Thermal conductivity of LM-D composites as a function of diamond content (1060 vol%); Figure S4. (a) SEM image of a 50 vol% LM-CCD composite after aging at 125 °C for 240 h; (b) enlarged view of the rectangular area shown in (a); Figure S5. EDS results for points 1–5 in Figure 3b; Figure S6. XRD results for the surface of a 50 vol% LM-CCD composite material following aging at 125 °C for 240 h (scan angle 10–80°, scan rate 2°/min); Figure S7. Practical application validation with an aluminium heat sink: (a) schematic diagram of the cooling system; (b) CPU temperature versus time curve; (c) comparison of steady-state temperature differences between the CPU and the heat sink; Table S1. EDS analysis results for points 1–5 in Figure 3b.
