# Supplementary material for: Study on the Thermal Properties and Reliability of Liquid Metal/Copper-Coated Diamond Composite Thermal Interface Materials
Source: Nanomaterials (Basel). 2026 Oct 22;16(21):1340. doi: 10.3390/nano16211340 (PMC13765070; doi:10.3390/nano16211340)
Supplement: Supplementary file 1 [file nanomaterials-16-01340-s001.zip › nanomaterials-4587057-supplementary.pdf]

Article

Study on the Thermal Properties and Reliability of Liquid Metal/Copper-coated Diamond Composite Thermal Interface Materials

Zimeng Chen [1], Bo Wang [1,*], Zhanglong Ke [1], Wei Huang [1], Yubing Gong [1], Siliang He [1,2,*], Kailin Pan [1]

[1] Guangxi Key Laboratory of Advanced Packaging and System Integration, School of Mechanical and Electrical Engineering, Guilin University of Electronic Technology, Guilin 541004, China; zimengc@163.com (Z.C.); 17827775516@163.com (Z.K.)
[2] Sun Yat-sen University Huizhou Research Institute, Huizhou 516081, China;
* Correspondence: wangbo_guet@163.com (B.W.); siliang_he@guet.edu.cn (S.H.)

Microscopic Morphology and Particle Size Analysis of CCD Particles

To accurately assess the particle size distribution, SEM images (Figure S1a) were processed using ImageJ software to extract the contours of individual particles (Figure S1b). Based on the extracted contours, the maximum and minimum Feret diameters (Feret, MinFeret) of each particle were calculated, and their geometric mean was taken as the equivalent particle size. Figure S2 shows the geometric mean particle size distribution for all particles analysed.

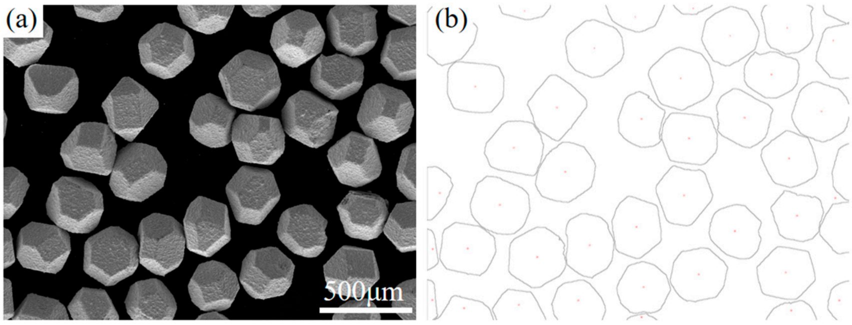

Figure S1. (a) Raw SEM morphology of CCD particles; (b) schematic diagram showing particle contour extraction and particle size measurement using ImageJ software.

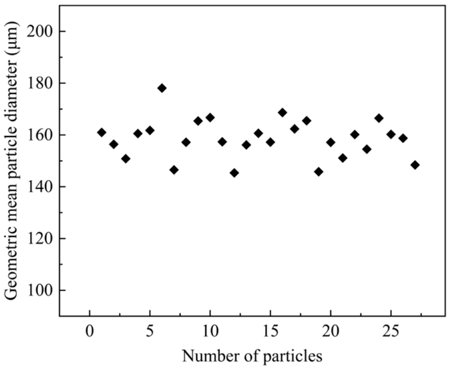

Figure S2. Particle-by-particle scatter plot of the geometric mean particle size of CCD particles.

Thermal Conductivity of LM-D Composite Materials

To evaluate the effect of filler content on the thermal conductivity of LM-D composites, samples with diamond contents of 10, 20, 30, 40, 50, and 60 vol% were prepared and tested under the same conditions as the LM-CCD composites. As shown in Figure S3, the thermal conductivity of the LM-D composites increased with increasing diamond content, reaching a maximum of approximately 73.5 W/(m·K) at 50 vol%, and then decreased at 60 vol%. This trend is similar to that of the LM-CCD composite, but the average thermal conductivity of the LM-D composite is lower than that of the LM-CCD composite at the same filler content, confirming the role of the copper coating in enhancing the thermal conduction network.

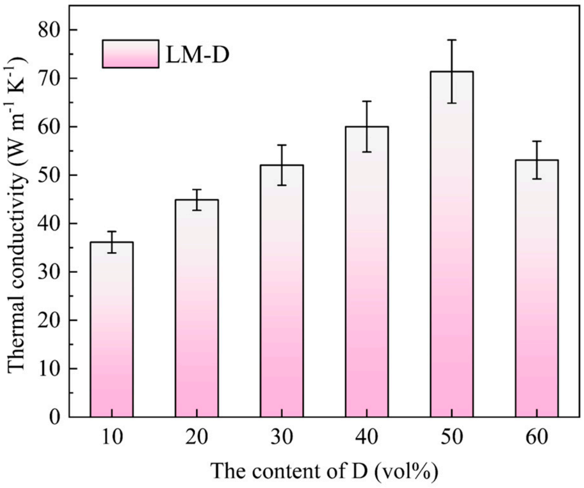

Figure S3. Thermal conductivity of LM-D composites as a function of diamond content (10-60 vol%).

Analysis of Reaction Products at the LM-CCD Composite Interface Following Aging

To further confirm the formation of $CuGa_2$ during high-temperature aging, a detailed analysis was carried out on the surface morphology and elemental composition of the 50 vol% LM-CCD composite after aging at 125 °C for 240 h. Figure S4 shows SEM images of the composite surface after aging, in which a large number of particles exposed on the material surface can be clearly observed. To identify the substances on the particle surfaces, five points (Points 1–5) were selected on these particles for EDS spot analysis; the corresponding EDS spectra are shown in Figure S5, and the quantitative results are summarised in Table S1. EDS analysis indicated that the precipitates consist primarily of Cu and Ga, with a Cu/Ga atomic ratio ranging from 0.45 to 0.65, which is close to the stoichiometric ratio of $CuGa_2$ (1:2). To further verify the phase composition, Benchtop X-ray Diffractometer, XRD, MiniFlex600-C, Rigaku, Japan) analysis was performed on the aged samples (Figure S6). The characteristic diffraction peaks showed good agreement with the standard PDF card for $CuGa_2$ (PDF#04-003-1650), confirming the formation of the tetragonal phase $CuGa_2$ during high-temperature aging. These results provide direct evidence for an interfacial reaction between Ga and Cu, which is the primary cause of microstructural degradation and the decrease in thermal conductivity in the LM-CCD composite material following aging.

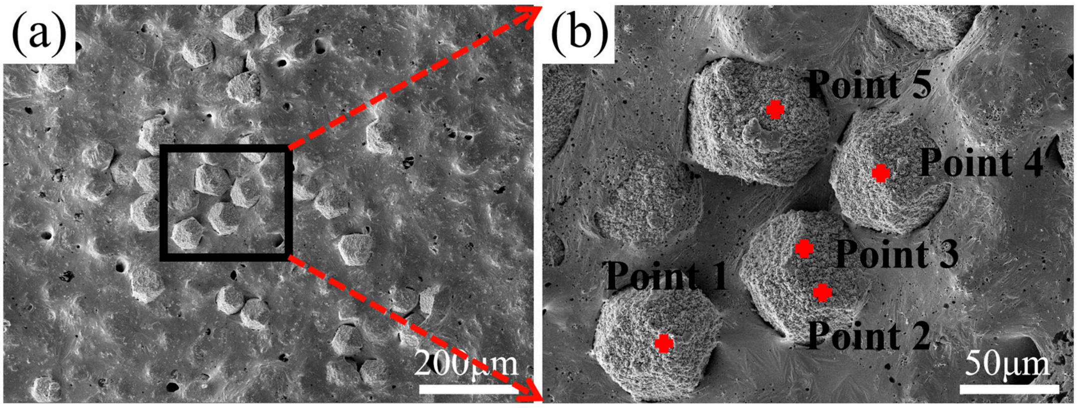

Figure S4. (a) SEM image of a 50 vol% LM-CCD composite after aging at 125 °C for 240 h; (b) enlarged view of the rectangular area shown in (a).

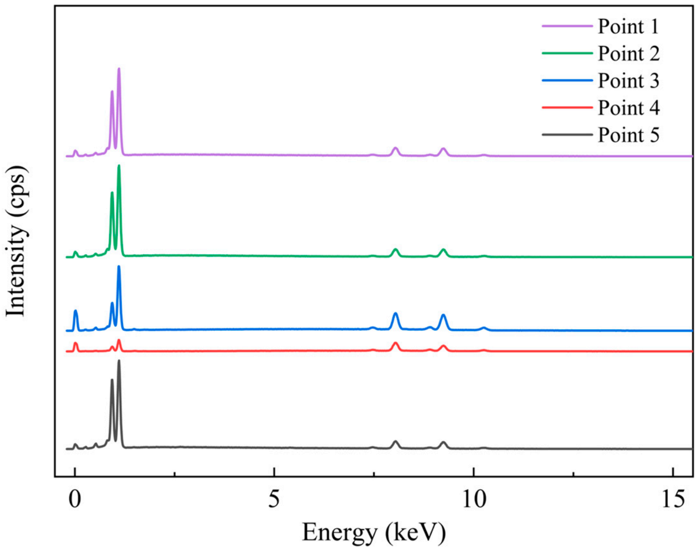

Figure S5. EDS results for points 1–5 in Figure 3(b).

Table S1. EDS analysis results for points 1–5 in Figure 3(b).

Element	Point 1 (At%)	Point 2 (At%)	Point 3 (At%)	Point 4 (At%)	Point 5 (At%)
Cu	32.41	39.28	31.40	30.89	31.61
Ga	67.59	60.72	68.60	69.11	68.39
Cu/Ga atomic ratio	0.48	0.65	0.46	0.45	0.46

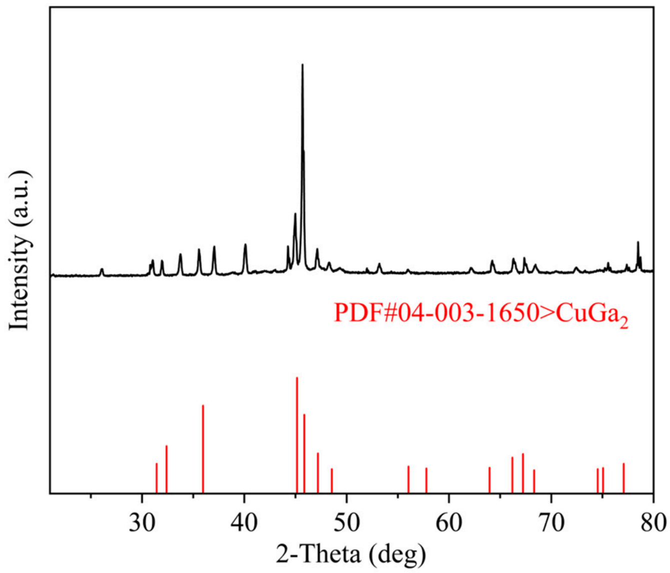

Figure S6. XRD results for the surface of a 50 vol% LM-CCD composite material following aging at 125 °C for 240 hours (scan angle 10–80°, scan rate 2°/min).

Field Testing of Aluminum Heat Sinks in Practical Applications

To compare the results with those of the copper heat sink presented in the main text, we also conducted raw, real-world application tests using an aluminum heat sink. Under identical assembly and operating conditions, we evaluated a commercial thermal grease and the LM-CCD composite material. Figure S7a shows a schematic diagram of the test system. Figure S7b shows the CPU temperature versus time curve, while Figure S7c compares the steady-state temperature difference between the CPU and the heat sink. These results are provided for comparison purposes only. It should be noted that, due to the risk of embrittlement, the use of aluminum in combination with gallium-based liquid metal thermal interface materials is not recommended.

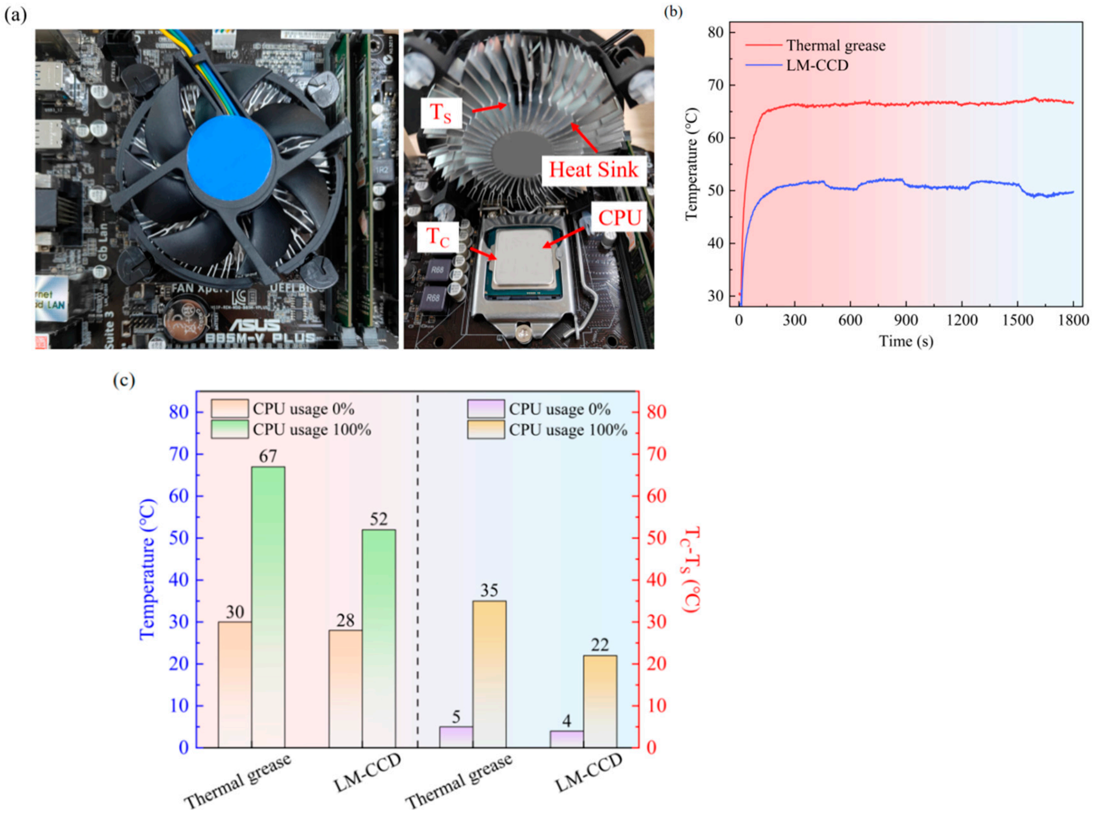

Figure S7. Practical application validation with an aluminium heat sink: (a) schematic diagram of the cooling system; (b) CPU temperature versus time curve; (c) comparison of steady-state temperature differences between the CPU and the heat sink.
